# Effect of Durum Wheat Oil on the Physico-Chemical and Sensory Features of Biscuits

**DOI:** 10.3390/foods11091282

**Published:** 2022-04-28

**Authors:** Francesca Vurro, Marcello Greco Miani, Carmine Summo, Francesco Caponio, Antonella Pasqualone

**Affiliations:** 1 Department of Soil, Plant and Food Science (DISSPA), University of Bari Aldo Moro, Via Amendola, 165/a, 70126 Bari, Italy; francesca.vurro@uniba.it (F.V.); carmine.summo@uniba.it (C.S.); francesco.caponio@uniba.it (F.C.); 2 Casillo Next Gen Food srl, Laboratory R&D Casillo Group, Via A. Sant’Elia, 70033 Corato, Italy; marcello.miani@casillogroup.it

**Keywords:** biscuits, durum wheat oil, by-products, tocotrienols, tocopherols, induction time, oxidation, sensory properties, volatile compounds

## Abstract

Lipids play an important role in defining the overall quality of biscuits, particularly in terms of resistance to oxidation, as well as for their influence on textural and sensorial properties. The aim of this work was to investigate the effects of durum wheat oil on the physico-chemical and sensory features of biscuits. Control biscuits (C) prepared with the commonly used sunflower oil were compared with samples prepared with durum wheat oil at 50% (D50) and 100% replacement levels (D100). The reformulated biscuits were very rich in tocols, especially tocotrienols (982.9, 635.2, and 64.1 mg/kg on lipid fraction weight in D100, D50, and C, respectively). The higher content of antioxidants extended the resistance to the oxidation of biscuits (induction time = 53.61, 70.87, and 79.92 h in C, D50, and D100, respectively). D100 showed the lowest amounts of triacylglycerol oligopolymers and oxidized triacylglycerols, and the lowest amounts of the volatile markers of lipid oxidation (hexanal and nonanal). The use of durum wheat oil did not affect the sensorial and textural properties, compared to C. This study suggests that durum wheat oil could be effectively used in biscuit-making to decrease the oxidative phenomena and increase the bioactives of the end-products.

## 1. Introduction

Biscuits are one of the most popular bakery items [1] and their global demand has grown by 31.6% during the outbreak of COVID-19 [2,3]. These products are widely consumed, due to their pleasant sensory characteristics, affordability, and long shelf-life [4,5].

The production process is relatively simple and consists of mixing the ingredients, shaping, baking, and packaging [6,7]. The usual ingredients are flour, fat or oil, sugar, water, and chemical leavening agents, and the optional minor ingredients are salt, eggs, emulsifiers, milk, and flavoring compounds [4,6,8]. Though not being perceived as fatty foods, biscuits show high contents of lipids, from 7.5% to 25% [9,10], which play an important technological role. During mixing, indeed, lipids lubricate flour and, due to their hydrophobic nature, inhibit hydration and gluten development [8]. As a consequence, lipids positively influence the physical and sensory characteristics of biscuits [11,12], conferring the typical ‘melt in the mouth’ and crumbly texture, as well as flavor [1,8]. Lipids, however, are susceptible to hydrolytic degradation, oxidation processes and thermal polymerization. The compounds originated by the oxidative degradation, namely triglyceride oligopolymers and oxidized triglycerides, are among the major biscuit contaminants related to storage [13] and are considered “nutritionally suspect”, with potential implications on human health [14]. Lipid oxidation causes the development of off-odors and flavors that negatively influence the palatability and shelf-life of biscuits. This aspect, together with the great impact on the texture, makes the choice of fat crucial [5,11,15].

The formulation of low-fat biscuits or the use of healthier fats represents a good opportunity for bakery companies to release new products [1]. In particular, vegetable oils or hydrocolloids and oleogels with lubricant and flow properties similar to those of fats have been proposed to replace them totally or partially [1,16,17,18]. Other studies proposed to replace fat with flour from tomato seeds, poppy seeds, or apricot kernels [19,20,21]. However, the problem of keeping the biscuit consistency unaltered remains of relevant importance, because fat replacement has a great impact on the textural attributes [8].

At a global level, wheat is the second cultivated cereal crop, with a production of over 775 million tons in 2021 and a similar production forecast for 2022 [22,23]. The majority is represented by soft wheat (*Triticum aestivum* L.), whereas durum wheat (*Triticum turgidum* L. var *durum* Desf.), which is fundamental in the production of pasta, bulgur, couscous, freekeh, and some types of bread [24,25,26], accounts for 5% [27]. Canada is the main producer of durum wheat globally, while Italy is the main producer in the European Union, contributing about 4 million tons [28]. The first Italian region for durum wheat production is Apulia, where more than 83% of the cereal-producing area is represented by this crop [29]. Germ, bran, and de-branning fractions are the main by-products of the wheat milling industry, mostly destined for animal feeding [30]. These by-products contain about 80% of the wheat lipids, roughly 65% in the germ, and 15% in bran, with a total lipid content accounting for 2.4–3.8% of wheat kernel weight on a dry basis [31]. The industrial extraction of oil from the wheat milling by-products is well established in the bread wheat chain, while the availability of durum wheat oil is still limited. This situation, however, is going to change because of ongoing investments—in particular in the Apulia region—aimed at exploiting the durum wheat milling by-products for extracting oil. Such investments have been prompted by the need of implementing the principles of the circular economy in the durum wheat chain [32]. A recent paper has evidenced the interesting nutritional properties of durum wheat oil, extracted from a 40/60 *w*/*w* mixture of milling and de-branning by-products (bran, germ, and de-branning fractions). Even after the refining process following the solvent extraction, this oil showed an outstanding content of phytosterols (20.9 g/kg; mainly composed of β-sitosterol, followed by sitostanol, campestanol, and campesterol) and policosanols (754 mg/kg) [33]. Furthermore, it showed a very high content of tocotrienols (about 1100 mg/kg) [33]. No studies, however, have considered the use of durum wheat oil in the preparation of biscuits so far.

After a campaign calling for the substitution of palm oil in bakery products, including biscuits with other oils, sunflower oil has become the most used in Italy. However, the very recent crisis in Ukraine, one of the major producers of sunflower oil (over 5 million tons in 2019) [22], caused a shortage of this kind of oil. Proposing a larger use in bakery products, namely biscuits, of the oil extracted from the by-products of durum wheat milling and de-branning could therefore: (i) significantly contribute to the expected transition from the linear economy to a circular economy in the durum wheat chain; (ii) improve the quality and nutritional value of the end-products; (iii) allow for a valid alternative in the case of shortage of other oils more frequently used.

The aim of this work was, therefore, to investigate the effects of durum wheat oil on the physico-chemical and sensory features of biscuits in comparison with sunflower oil.

## 2. Materials and Methods

### 2.1. Materials

Wheat flour type 00 (Despar Italia, Casalecchio di Reno, Italy) (carbohydrates 72 g/100 g; protein 10 g/100 g; fat 1.7 g/100 g; fiber 1.4 g/100 g), sugar (sucrose) (Despar Italia, Casalecchio di Reno, Italy), partially skimmed milk (Granarolo, Bologna, Italy) (carbohydrates 5 g/100 g, protein 3.4 g/100 g; fat 1.6 g/100 g), baking powder (R. Barra s.a.s., Crispiano, Italy), refined sunflower oil (Olearia De Santis, Bitonto, Italy) were purchased from local retailers. Durum wheat oil, produced as reported in Squeo et al. [33], was provided by Casillo Next Gen Food srl (Corato, Italy).

### 2.2. Biscuit Preparation

Three biscuit types were prepared with: 100% sunflower oil (C); 50% sunflower oil and 50% durum wheat oil (D50); and 100% durum wheat oil (D100), according to the formulation reported in Table 1. Biscuit formulation was defined by means of preliminary trials. The process consisted of (i) mixing wheat flour, sugar, and oil using a spiral kneader (Bosh MFQ40304, München, Germany) for 5 min; (ii) adding partially skimmed milk and baking powder; and kneading for 12 min; (iii) rolling the dough and shaping as rectangular biscuits (6 cm length; 2.5 cm width; 1 cm thickness); and (iv) baking in an electric oven (Smeg SI 850 RA-5 oven, Smeg S.p.A., Guastalla, Italy) for 16 min at 160 °C. Biscuits were placed in the baking tray according to a randomized block distribution to take into account possible border effects.

### 2.3. Determination of the Resistance to Oxidation

The resistance to oxidation was determined by RapidOxy (Anton Paar, Blankenfelde-Mahlow, Germany). The samples (1 g) were analyzed at 140 °C and under 700 kPa O_2_ pressure. The induction time, i.e., the time needed for a 10% drop of the O_2_ pressure, was recorded. The analysis was carried out in triplicate.

### 2.4. Determination of Tocopherols and Tocotrienols

The tocopherols and tocotrienols of the oils and of the fatty fraction of biscuits—the latter extracted by Soxhlet method, using diethyl ether (SER 148 extraction system, Velp Scientifica srl, Usmate, Italy)—were determined by RP-UHPLC-FLD (Dionex Ultimate 3000 RSLC, Waltham, MA, USA). In particular, 0.02–0.03 g of sample was dissolved in 1 mL of 2-propanol. The samples were filtered by a 0.45 µm polytetrafluoroethylene (PTFE) filter and injected into a UHPLC system consisting of an HPG-3200 RS Pump, a WPS-3000 autosampler, a TCC-3000 column compartment, and an FLD-3400RS fluorescent detector (excitation wavelength 295 nm, emission wavelength 325 nm). The stationary phase was a Dionex Acclaim 120 C18 analytical column (Thermo Scientific, Waltham, MA, USA) with 3 µm particle size, 120 Å, 3 × 150 mm; the mobile phase was 1:1 (*v*/*v*) methanol and acetonitrile at a flow rate of 1 mL/min in isocratic elution. The software was Chromeleon (Dionex-ThermoFisher Scientific, Waltham, MA, USA). The single tocopherols were determined by external standard method on the basis of a previously set calibration curves obtained for α-tocotrienols and α-tocopherols. The content of tocopherols and tocotrienols were expressed as mg/kg on lipid fraction weight. The determinations were carried out in triplicate.

### 2.5. Determination of Polar Compounds of the Lipid Fraction

The polar compounds were recovered from the lipid fraction of biscuits by silica gel column chromatography and analyzed by high-performance size-exclusion chromatography (HPSEC) as described by Caponio et al. [34] with the only modification of using tetrahydrofuran (THF) as eluant, instead of dichloromethane. The analyses were carried out in duplicate.

### 2.6. Determination of Volatile Compounds

The volatile compounds of biscuits were determined by headspace solid-phase micro-extraction (HS-SPME) coupled with gas chromatography/mass spectrometry (GC–MS) as previously reported [35]. The quantification was carried out by standardizing the peak areas of the volatile compounds with the peak area of the internal standard (1-propanol). The analyses were carried out in duplicate.

### 2.7. Texture Profile Analysis

The textural properties of biscuits were determined by a 3-point bending test as described in Pasqualone et al. [35], with few modifications. The force (N) required to fracture the sample was recorded as biscuit hardness. A Texture Analyzer (Z1.0 TN, Zwick GmbH & Co., Ulm, Germany), equipped with a 1000 N load cell, was used. The distance between the support bars was 3 cm. The probe, the speed of which was set at 5 mm/min, moved downward until the biscuit was broken. Six replicated analyses were carried out.

### 2.8. Color Measurement

The color of the biscuits was analyzed in the CIE L*a*b* scale, under a D65 illuminant, by using a CM-600d colorimeter (Konica Minolta, Tokyo, Japan). Lightness (L*), redness (a*), and yellowness (b*) were measured. Ten replicated analyses were carried out.

The total color variation (∆E) was calculated to compare the differences between C biscuits and the two types of biscuits containing durum wheat oil (D50 and D100), according to the equation:

$$\Delta E=[{\left(L^{*}-L_{0}^{*}\right)}^{2}+{\left(a^{*}-a_{0}^{*}\right)}^{2}+{\left(b^{*}-b_{0}^{*}{)}^{2}\right]}^{1/2}$$

where $L_{0}^{*}$, $a_{0}^{*}$, and $b_{0}^{*}$ were the color coordinates for the reference biscuits (C), whereas L*, b*, a* were the color coordinates of the other samples. The mean values were considered in the calculation. The obtained results were then evaluated according to the following ΔE scale: 0–2.0 = unrecognizable difference; 2.0–3.5 = difference recognizable by an experienced observer; <3.5 = clear difference [36].

### 2.9. Determination of Dimensional Parameters

The dimensional parameters of biscuits (thickness, width, and length) were measured before and after baking by a calliper, and the increase induced by baking was calculated by difference. Six replicated analyses were carried out.

### 2.10. Determination of Sensory Properties

The quantitative descriptive analysis (QDA) of the sensory properties of biscuits was carried out according to the International Standardization Organization (ISO) standard 13299 [37], by a trained panel of 8 members. Panelists (4 men and 4 women) ranged in age from 23 to 55 years. Panelists, regular consumers of biscuits, had neither food allergies nor intolerances. They were informed about the study aims and provided written consent to perform the sensory analysis, according to the ethical guidelines of the laboratory of Food Science and Technology of the Department of Soil, Plant and Food Science of the University of Bari (Italy). Pre-test sessions were made to define the list of descriptors and to verify the discriminating ability, consistency, and reliability of panelists, as in the ISO Standard 11132 [38]. The sensory terms are defined in detail in Table 2. The intensity of every attribute was expressed on a 10 cm unstructured linear scale. The samples were randomized and presented to the panelists in white dishes marked with alphanumeric codes. The sensory properties were evaluated in a conference room, where temporary partitions were used to set up isolated tasting booths for separating the panelists during the analysis, in agreement with the ISO standard 8589 [39]. The testing was performed at ambient room temperature (20 ± 2 °C).

### 2.11. Statistical Analysis

The results were expressed as the mean ± standard deviation (SD). Significant differences were determined at *p* < 0.05, according to the one-way analysis of variance (ANOVA), followed by a Tukey test for multiple comparisons. Statistical analysis was carried out by the Minitab Statistical Software (Minitab Inc., State College, PA, USA).

## 3. Results and Discussion

### 3.1. Tocopherols and Tocotrienols Content, Resistance to the Oxidation, and Polar Compounds

Table 3 shows the content of tocopherols and tocotrienols in the oils used in the experimental trials, and in the obtained biscuits. Durum wheat oil was remarkably rich in tocotrienols, which instead were assessed in very low amounts in sunflower oil. On the contrary, significantly higher levels of tocopherols were observed in sunflower oil than in the durum wheat one. Overall, the total sum of tocopherols + tocotrienols in durum wheat oil accounted for 1425.2 mg/kg, roughly doubling the total amount of sunflower oil. The wheat germ, which was present in the by-product mixture used to extract the durum wheat oil used in these biscuit-making trials, is known to be an important source of tocopherols and tocotrienols. These compounds are a group of eight isomers, collectively known as tocols or vitamin E, synthesized only by plants and photosynthetic microorganisms [40]. The different forms of tocopherols and tocotrienols (α, β, γ, δ) depend on the number and location of methyl groups in the hydrophilic head of 6-chromanol [40,41]. Tocopherols and tocotrienols both act as natural antioxidants [42,43,44]. Furthermore, tocotrienols have been reported to be effective in the prevention of cancer-related processes, cardiovascular pathologies, and Alzheimer’s disease [45,46,47].

The different concentrations of tocopherols and tocotrienols of the two oils influenced the content of these compounds in biscuits: those prepared with 100% durum wheat oil showed a higher tocotrienol concentration and lower tocopherol level than biscuits prepared with total or partial replacement of sunflower oil. The content of tocotrienols observed in all the biscuits was also positively influenced by the contribution of wheat flour, known to contain more tocotrienols than tocopherols [48,49], thus explaining the presence of tocotrienols observed in the 100% sunflower oil-containing biscuits.

Table 4 reports the resistance to the oxidation of the oils and the experimental biscuits. The results are expressed as induction time (IT), i.e., the ‘stability time’ before fat oxidation, which corresponds to a 10% decrease of the O_2_ pressure in the testing device due to the consumption of oxygen by the sample being oxidized [50].

Durum wheat oil was more stable than sunflower against the onset of rancidity and oxidative deterioration, as indicated by its higher value of IT. The analysis of biscuits evidenced that replacing sunflower oil with durum wheat oil progressively and significantly increased the IT according to the percentage of replacement. This result was due to the high level of total tocols of the durum wheat oil. Similarly, Sharif et al. [51] observed that biscuits prepared with rice bran oil had an extended shelf-life due to high levels of tocopherols, tocotrienols, and oryzanols.

To have a better insight into the effect of oil substitution on biscuit oxidation, which starts during the production process and goes ahead during storage [13], the analysis of polar compounds was also carried out (Table 5). This analysis enabled the separation and quantification of the different classes of substances due to both oxidation (triacylglycerol oligopolymers and oxidized triacylglycerols) and hydrolysis (diacylglycerols) of any lipid [9,52]. The oxidation products, in particular, are most suspected of altering the nutritional properties of foods and causing adverse physiological effects [14,53,54]. The biscuits prepared with sunflower oil showed significantly higher contents of triacylglycerol oligopolymers and oxidized triacylglycerols than biscuits with durum wheat oil. This result was in line with the lower level of antioxidants observed in the former.

The biscuits prepared with durum wheat oil, however, showed a higher level of lipid hydrolytic degradation than biscuits made with sunflower oil, mirroring the high content of diacylglycerols of the durum wheat oil used [33]. The latter, in turn, was probably due to lipolytic phenomena occurred in the starting wheat milling/de-branning by-products, against which containment measures should be taken. On the other hand, the most detrimental for quality are the compounds derived from the oxidative degradation. In a comparative study involving the use of different oils (extra virgin olive oil, olive oil, olive-pomace oil, and refined palm oil) in the preparation of dry bakery products similar to biscuits [55], it appeared that the choice of lipid was very influential on quality because refined oils showed high levels of oxidized triacylglycerols and polymerization compounds, which further raised during processing. Similarly, the levels of lipid degradation compounds ascertained in an early survey on the quality of Italian biscuits, where refined oils and margarines were mostly used, were found to be high [9]. On the contrary, durum wheat oil, despite the detrimental effect of the refining process [33], is rich in antioxidants which help limiting the formation of the polar compounds, particularly the oxidation-related ones.

### 3.2. Volatile Compounds

The volatile compounds were significantly different among biscuits (Table 6). In particular hexanal, hexenal, 2-heptenal, nonanal were lower in biscuits with durum wheat oil than in the biscuits with sunflower oil. These compounds are related to the typical rancid off-flavor and are considered markers of lipid oxidation [56]. Hexanal derives from the oxidation of linoleic acid, while hexenal comes from the linolenic acid. In general, the aldehydes deriving from the action of lipoxygenase are responsible for undesirable odors, especially hexanal. The lower presence of these compounds in the biscuits prepared with the durum wheat oil indicated a lower level of oxidation and was in line with the ascertained levels of polar compounds. The high level of tocols present in the durum wheat oil reduced the oxidation therefore limiting the presence of volatile oxidation markers in biscuits. This was in agreement with Kishimoto et al. [57], who observed that α-tocopherol added to extra virgin olive oil reduced the formation of hexanal and other oxidative markers during storage.

2-Methylbutanal and 3-methylbutanal were both more abundant in the biscuits prepared with durum wheat oil. They are Strecker aldheydes characterized by a malty odor, derived by the reaction of Maillard from the aminoacids isoleucine and leucine, respectively. Benzaldehyde, derived from the phenylalanine metabolism [58], showed the same trend. The Maillard reaction also determined the formation of pyrazines and furan compounds. Though the carboxen/polydimethylsiloxane SPME fiber used is not very sensitive to pyrazine, three of them were identified: pyrazine, methyl-pyrazine, and ethyl-pyrazine. They were significantly more abundant in biscuits with durum wheat oil than in those prepared with sunflower oil. In addition, 2-furanmethanol and 2-furancarboxaldehyde (or furfural), the latter being commonly detected in biscuits [35], were assessed in higher amounts in the biscuits with durum wheat oil.

### 3.3. Physical Characteristics

**Figure 1:**
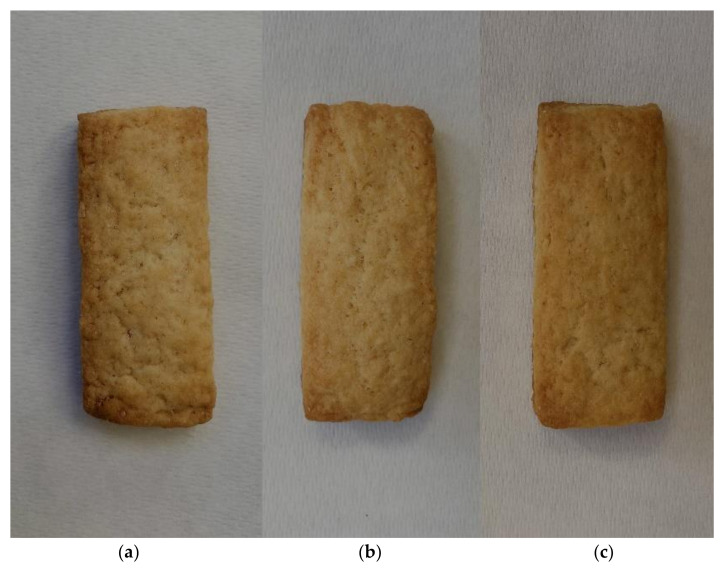
(**a**) Biscuits prepared with 100% sunflower oil; (**b**) biscuits prepared with sunflower oil (50%) and durum wheat oil (50%); (**c**) biscuits prepared with 100% durum wheat oil.

Table 7 shows the physical characteristics of the experimental biscuits (color, texture, and dimensional variations during baking). Replacing the sunflower oil with the durum wheat oil did not determine statistically significant changes in the color coordinates a* (redness), b* (yellowness), and L* (luminosity). Therefore, the color difference (∆E) between the reference biscuits (C) and those containing durum wheat oil (D50 and D100) was unrecognizable (values lower than 2). All biscuits showed a similar golden-brown color (Figure 1), imputable to Maillard reaction and caramelization. These reactions develop the typical color, as well as intense flavor and taste, very important in baked goods [8].

Biscuit hardness was determined by fracturing the samples through a three-point bending test. Biscuits prepared with sunflower oil were significantly (*p* < 0.05) harder than those obtained by using exclusively durum wheat oil. The increased presence of diacylglycerols in the latter could have determined an easier breakability. Mono- and diacylglycerols are, indeed, commonly used in the bakery sector as emulsifiers [59,60] to ensure proper gas retention during dough mixing and produce a more aerated structure, which in biscuits means a less hard consistency [61]. Biscuit hardness has an influence on consumer acceptability, with less hard biscuits being easier to chew and more appreciated. Other authors, in reformulating biscuits to improve their nutritional quality, added softening ingredients, such as honey or oleogels, to avoid excessive hardness [62,63].

The dimensional increase, caused by the thermal expansion of gases during baking [64], was not found to be significantly different when the oil type changed. The absence of significant variations as a consequence of the oil substitution can be explained being sunflower and durum wheat oil similar in lubricant ability and flow properties. The biscuits increased more in thickness than in length and width, as observed in other studies [56,64].

### 3.4. Sensory Features

The sensory properties did not show significant differences among the examined biscuits, with the only exception of breakability (Table 8). A significantly more pronounced breakability of biscuits with durum wheat oil was indeed observed, compared to the samples containing sunflower oil at 50% and 100%. These findings were in agreement with the instrumental measurement of hardness, which is inversely related to breakability. All biscuits were finely porous and showed an intense typical shortbread odor. Only a mild caramel odor was perceived, while no oxidized oil odor was perceived.

## 4. Conclusions

Results from the study demonstrated that durum wheat oil incorporation into biscuits improves the oxidative stability of the end-products, due to the high content of tocotrienols which characterizes this kind of oil. Moreover, the use of durum wheat oil did not influence negatively the physical and sensory characteristics of biscuits compared to the commonly used sunflower oil.

Wheat oil is currently produced mostly from soft wheat germ by solvent extraction and subsequent refining, being the by-products from durum wheat de-branning and milling still largely underused. The food industry should start exploiting its full potential, still partially undiscovered [65], also to face the possible unavailability of soft wheat. Innovative uses of durum wheat by-products would lead to an improvement of the nutritional characteristics of food products and satisfy customers’ demands for healthy and functional foods. Further investigations will be therefore carried out to study the use of durum wheat oil in other bakery products.

## Figures and Tables

**Table 1:** Formulation of the experimental biscuits. C = Biscuits prepared with 100% sunflower oil; D50 = biscuits prepared with sunflower oil (50%) and durum wheat oil (50%); D100 = biscuits prepared with 100% durum wheat oil.

Ingredients	C (g)	D50 (g)	D100 (g)
Wheat flour	400	400	400
Sunflower oil	112	56	−
Durum wheat oil	−	56	112
Sugar	112	112	112
Partially skimmed milk	128	128	128
Baking powder	4.8	4.8	4.8

**Table 2:** Descriptive terms used for the sensory profiling of biscuits.

Descriptor	Definition	Scale Anchors	
		Min = 0 (c.u.) *	Max = 10 (c.u.)
Visual–tactile characteristics			
Porosity	Presence of pores	Absent	Very intense
	The way the biscuit fractures when broken by fingers	Breaks with difficulty	Crumbly, breaks easily
Breakability	The way the biscuit fractures when broken by fingers	Breaks with difficulty	Crumbly, breaks easily
Odor notes			
Caramel	Typical odor associated with caramel	Absent	Very intense
Oxidized oil	Typical odor associated with oxidized oil	Absent	Very intense
Shortbread	Typical odor associated with biscuits	Absent	Very intense

* c.u. = contractual units.

**Table 3:** Total tocopherols and tocotrienols of the oils and biscuits. C = Biscuits prepared with 100% sunflower oil; D50 = biscuits prepared with sunflower oil (50%) and durum wheat oil (50%); D100 = biscuits prepared with 100% durum wheat oil.

Sample	Tocopherols (mg/kg)	Tocotrienols (mg/kg)
*Oils*		
Sunflower oil	677.9 ± 7.1 ^a^	7.4 ± 1.4 ^b^
Durum wheat oil	305.6 ± 7.6 ^b^ **	1119.6 ± 19.5 ^a^ **
*Biscuits*		
C	601.8 ± 10.1 ^a^	64.1 ± 11.8 ^c^
D50	418.9 ± 11.1 ^b^	635.2 ± 38.7 ^b^
D100	280.6 ± 8.3 ^c^	982.9 ± 11.2 ^a^

** From [33]. Different letters in the same column, for the same sample type, indicate significant differences at *p* < 0.05.

**Table 4:** Resistance to oxidation of the oils and biscuits. C = Biscuits prepared with 100% sunflower oil; D50 = biscuits prepared with sunflower oil (50%) and durum wheat oil (50%); D100 = biscuits prepared with 100% durum wheat oil.

Sample	IT * (h)
*Oils*	
Sunflower oil	31.50 ± 0.42 ^b^
Durum wheat oil	39.80 ± 0.09 ^a^
*Biscuits*	
C	53.61 ± 1.87 ^c^
D50	70.87 ± 2.94 ^b^
D100	79.92 ± 2.21 ^a^

* IT = Induction time. Different letters for the same sample type indicate significant differences at *p* < 0.05.

**Table 5:** Polar compounds of the lipid fraction of the experimental biscuits. C = Biscuits prepared with 100% sunflower oil; D50 = biscuits prepared with sunflower oil (50%) and durum wheat oil (50%); D100 = biscuits prepared with 100% durum wheat oil.

Compound (g/kg)	Sample Type		
	C	D50	D100
TAGP	0.43 ± 0.03 ^a^	0.31 ± 0.02 ^b^	0.17 ± 0.02 ^c^
ox-TAG	3.52 ± 0.13 ^a^	2.63 ± 0.43 ^b^	1.81 ± 0.19 ^b^
DG	1.30 ± 0.02 ^c^	3.03 ± 0.05 ^b^	4.93 ± 0.17 ^a^

TAGP = triacylglycerol oligopolymers; ox-TAG = oxidized triacylglycerols; DG = diacylglycerols Different letters in the same row indicate significant differences at *p* < 0.05.

**Table 6:** Volatile compounds of biscuits. C = Biscuits prepared with 100% sunflower oil; D50 = biscuits prepared with sunflower oil (50%) and durum wheat oil (50%); D100 = biscuits prepared with 100% durum wheat oil.

Volatile Compounds (µg/g)	Sample Type		
	C	D50	D100
*Aldehydes*			
Hexanal	40.88 ± 0.49 ^a^	33.53 ± 0.26 ^b^	25.97 ± 1.06 ^c^
Hexenal	5.38 ± 0.35 ^a^	2.20 ± 0.11 ^b^	2.52 ± 0.31 ^b^
2-Heptenal	4.69 ± 0.17 ^a^	2.83 ± 0.03 ^b^	0.42 ± 0.16 ^c^
Nonanal	11.83 ± 0.61 ^a^	6.36 ± 0.36 ^b^	6.38 ± 0.22 ^b^
2-Methylbutanal	10.26 ± 0.23 ^b^	13.92 ± 1.42 ^a^	16.88 ± 0.41 ^a^
3-Methylbutanal	17.71 ± 0.86 ^b^	25.41 ± 0.77 ^a^	27.03 ± 0.89 ^a^
Benzaldehyde	4.85 ± 0.38 ^b^	7.12 ± 0.92 ^a^	7.24 ± 0.15 ^a^
*Furan compounds*			
2-Furanmethanol	4.60 ± 0.51 ^b^	8.78 ± 0.74 ^a^	9.95 ± 0.68 ^a^
2-Furancarboxaldehyde (furfural)	3.67 ± 0.25 ^b^	5.41 ± 1.99 ^ab^	8.60 ± 0.44 ^a^
*Pyrazines*			
Pyrazine	8.27 ± 1.19 ^b^	13.10 ± 0.13 ^a^	13.15 ± 0.20 ^a^
Methyl-pyrazine	30.79 ± 1.31 ^b^	30.90 ± 1.82 ^b^	44.19 ± 1.71 ^a^
Ethyl-pyrazine	7.08 ± 1.21 ^b^	6.15 ± 0.48 ^b^	10.53 ± 0.17 ^a^

Different letters in the same row indicate significant differences at *p* < 0.05.

**Table 7:** Physical characteristics (color, texture, and dimensional variations during baking) of the experimental biscuits. C = Biscuits prepared with 100% sunflower oil; D50 = biscuits prepared with sunflower oil (50%) and durum wheat oil (50%); D100 = biscuits prepared with 100% durum wheat oil.

Parameter	Sample Type		
	C	D50	D100
Color			
a*	6.20 ± 0.32 ^a^	7.04 ± 0.68 ^a^	7.04 ± 0.52 ^a^
b*	33.33 ± 1.51 ^a^	32.47 ± 0.30 ^a^	34.56 ± 1.40 ^a^
L*	74.16 ± 1.63 ^a^	73.96 ± 0.52 ^a^	73.37 ± 1.43 ^a^
∆E	-	1.23	1.46
Texture			
Hardness (N)	19.10 ± 0.45 ^a^	18.70 ± 0.51 ^a^	16.62 ± 0.53 ^b^
Dimensional variations during baking			
Thickness increase (mm)	7.5 ± 0.5 ^a^	7.0 ± 0.5 ^a^	7.2 ± 0.6 ^a^
Length increase (mm)	1.5 ± 0.1 ^a^	1.2 ± 0.3 ^a^	1.5 ± 0.1 ^a^
Width increase (mm)	2.0 ± 0.1 ^a^	1.5 ± 0.5 ^a^	1.7 ± 0.6 ^a^

Different letters in the same row indicate significant differences at *p* < 0.05.

**Table 8:** Sensory features of the experimental biscuits. C = Biscuits prepared with 100% sunflower oil; D50 = biscuits prepared with sunflower oil (50%) and durum wheat oil (50%); D100 = biscuits prepared with 100% durum wheat oil.

Sensory Descriptor (c.u.) *	Sample Type		
	C	D50	D100
Visual–tactile characteristics			
Porosity	4.2 ± 0.4 ^a^	4.4 ± 0.2 ^a^	5.2 ± 0.5 ^a^
Breakability	3.5 ± 0.2 ^b^	4.3 ± 0.4 ^a^	5.1 ± 0.3 ^a^
Odor notes			
Caramel	0.6 ± 0.2 ^a^	0.7 ± 0.2 ^a^	0.7 ± 0.1 ^a^
Oxidized oil	0.1 ± 0.1 ^a^	0.0 ± 0.0 ^a^	0.0 ± 0.0 ^a^
Shortbread	7.6 ± 0.5 ^a^	7.3 ± 0.5 ^a^	6.6 ± 0.7 ^a^

* c.u. = contractual units. Different letters in the same row indicate significant differences at *p* < 0.05.

## Data Availability

The data presented in this study are available upon request from the corresponding author. The data are not publicly available due to the absence of in institutional platform.
